# Comparison of the early effects of vonoprazan, lansoprazole and famotidine on intragastric pH: a three-way crossover study

**DOI:** 10.3164/jcbn.17-128

**Published:** 2018-05-09

**Authors:** Kanji Ohkuma, Hiroshi Iida, Yumi Inoh, Kenji Kanoshima, Hidenori Ohkubo, Takashi Nonaka, Koji Fujita, Akihiko Kusakabe, Masahiko Inamori, Atsushi Nakajima

**Affiliations:** 1 Department of Gastroenterology and Hepatology, Yokohama City University School of Medicine, 3-9 Fukuura, Kanazawa-ku, Yokohama 236-0004, Japan; 2 Department of Medical Education, Yokohama City University School of Medicine, 3-9 Fukuura, Kanazawa-ku, Yokohama 236-0004, Japan; 3 Department of General Medicine, Yokohama City University School of Medicine, 3-9 Fukuura, Kanazawa-ku, Yokohama 236-0004, Japan

**Keywords:** intragastric acidity, vonoprazan, lansoprazole, famotidine

## Abstract

To promote symptom relief from acid-related diseases, a medicine with a rapid-onset effect is ideal. The aim of this study was to investigate the early inhibitory effect on gastric acid secretion after a single oral administration of vonoprazan, which represents a new class of proton pump inhibitors, and to compare this effect with those of lansoprazole and famotidine. Ten *Helicobacter pylori* (*HP*)-negative male subjects participated in this randomized, three-way crossover study. A single oral administration of vonoprazan (20 mg), lansoprazole (30 mg) or famotidine (20 mg) was performed, and the intragastric pH was continuously monitored for 6 h. Each drug was administered at least seven days apart. The average intragastric pH during the 6-h period after the administration of famotidine was higher than that after the administration of lansoprazole (median: 4.45 vs 2.65; *p* = 0.0284). A similar result was observed for vonoprazan and lansoprazole (median: 4.30 vs 2.65; *p* = 0.0322). In conclusions, oral administration of vonoprazan and famotidine in *HP*-negative healthy male subjects caused the intragastric pH to rise more quickly than did lansoprazole. (Trial Registration: UMIN000020989)

## Introduction

Recently, the number of Japanese patients with gastro-oesophageal reflux disease (GERD) has increased due to changes in eating habits and a decrease in *Helicobacter pylori* (*HP*) infections. In Japan, heartburn is a pervasive problem in Japan that interferes with daily life. Therefore, alleviating symptoms must be the primary objective in the treatment of symptomatic GERD patients.^(1)^ Proton pump inhibitors (PPIs) are widely used worldwide as a treatment for acid-related diseases (such as GERD and peptic ulcer disease) and a component of eradication therapy for *HP*.^(2–5)^ However, acid-suppressive therapy with PPIs has several limitations. Most GERD patients can be managed with standard PPI regimens; approximately 10–40% of patients have refractory symptoms.^(6)^ For patients with acid-related diseases, rapid onset of a drug that leads to the alleviation of symptoms is important.

Potassium-competitive acid blockers (P-CABs) are a new class of gastric acid suppressants. Similarly to PPIs, P-CABs inhibit gastric hydrogen/potassium adenosine triphosphatase (H^+^/K^+^-ATPase); however, dissimilarly to PPIs, P-CABs inhibit K^+^ competitively and reversibly.^(7)^ Vonoprazan is a new orally active P-CAB that was discovered and synthesized by Takeda Pharmaceutical Co., Ltd., Japan. Vonoprazan accumulates in the gastric tissue and remains for a long period; consequently, it has a long-lasting and potent anti-secretory effect on H^+^/K^+^-ATPase.^(8)^

To date, there have been few studies directly comparing the effects of vonoprazan and PPIs. Sakurai *et al.*^(9)^ reported that the acid-inhibitory effect (pH 4 holding time ratio) of vonoprazan is significantly greater than that of PPIs. Our previous study demonstrated that H_2_-receptor antagonists increase the intragastric pH faster than PPIs during the early post-administration phase.^(10–12)^ No study has directly compared the intragastric pH of vonoprazan (a new class of PPIs), lansoprazole (a conventional PPI) and famotidine (an H_2_-receptor antagonist; H_2_RA). Thus, we designed a three-way crossover study to compare the early effects of vonoprazan, lansoprazole and famotidine by using pH monitoring.

## Materials and Methods

### Subjects

We performed a randomized, three-way crossover study. It was conducted in 10 healthy male volunteers, aged between 20 and 37 years (mean age of 26.9 years) who did not take gastric acid secretion inhibitors such as H2Ras and/or PPIs. Prior to the study, we tested anti-*HP* immunoglobulin G (IgG) antibodies (SRL, Tokyo, Japan), and all subjects were negative.

### Study protocol and pH measurement

In this three-way crossover study, all subjects received a single oral dosage of vonoprazan (20 mg) (Takecab^®^, Takeda Pharmaceutical Co., Ltd.), a single oral dosage of lansoprazole (30 mg) (Takepron^®^, Takeda Pharmaceutical Co., Ltd.) and a single oral dosage of famotidine (20 mg) (Gaster^®^, Astellas Pharmaceutical Co., Ltd., Japan) in a crossover manner with a random sequence. Each of these drugs was administered for more than seven days. The subjects were requested to fast on the night before treatment (at least 8 h) and for a period of 6 h after drug administration. All medicines were given in the morning.

Under local anaesthesia, the subjects were nasally inserted with the pH electrode. The electrode tip was placed in the stomach. The intragastric pH was measured at 10-s intervals by a portable pH meter equipped with an antimony pH electrode (Chemical Instrument Co. Ltd.). Prior to each study, the pH electrode was calibrated by using standard buffer solutions of pH 4.01 and 6.86. The obtained pH data were analysed using computer software (Chemical Instrument Co., Ltd.). We measured the average pH, the average pH per hour, and the pH holding time at 6 h after each drug administration.

### Statistical analysis

We used Wilcoxon’s signed-rank test for the data. The significance level was set at a *p* value of <0.05. All statistical analyses used the StatView program (SAS Institute, Cary, NC).

### Ethics

This study was enforced according to the Declaration of Helsinki. The Yokohama City University Medical School Ethics Committee approved the study protocol and received written informed consent from all participants. This study is registered to the University Hospital Medical Information Network (UMIN) clinical trials registry (UMIN000020989). All authors had access to the clinical data and approved the final version of the manuscript.

## Results

### Adverse events

All 10 volunteers completed the study protocol, and adverse events were not reported.

### Average pH

**Fig. 1:**
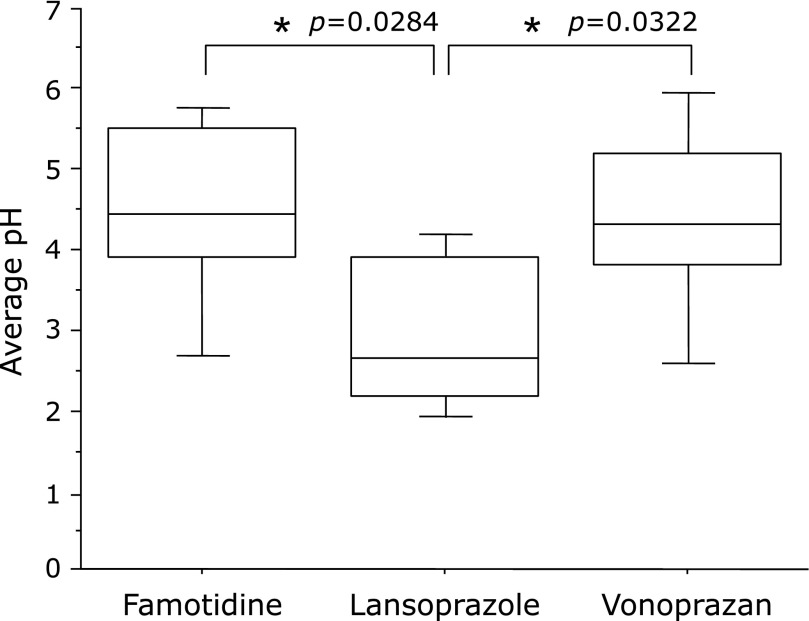
The average pH during the first 6 h was higher after the administration of famotidine and vonoprazan than after lansoprazole. *p<0.05 according to the Wilcoxon signed-ranks test.

The average pH during the 6-h period after administration was significantly higher with famotidine than with lansoprazole (median: 4.45 vs 2.65; *p* = 0.0284). Similar results were found for vonoprazan and lansoprazole (median: 4.30 vs 2.65; *p* = 0.0322). No significant differences were found between famotidine and vonoprazan (median: 4.45 vs 4.30; *p* = 0.7581) (Fig. 1).

**Fig. 2:**
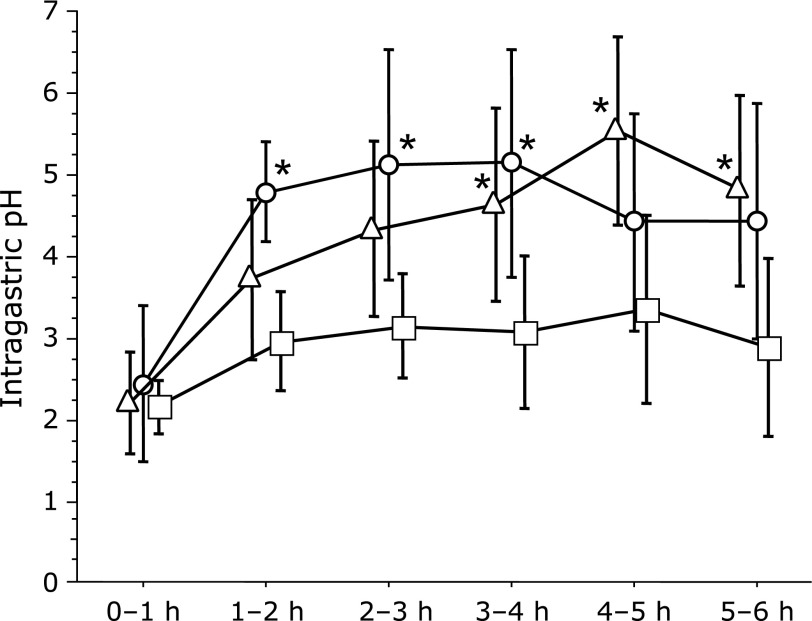
Famotidine (20 mg) resulted in a higher average pH than did lansoprazole (30 mg) in the 1–2, 2–3 and 3–4 h study periods after administration. Vonoprazan (20 mg) resulted in a higher average pH than did lansoprazole (30 mg) in the 3–4, 4–5 and 5–6 h study periods after administration. Circles (famotidine), triangles (vonoprazan) and squares (lansoprazole), mean values; vertical lines, SD; horizontal line, ±SD. *p<0.05 according to the Wilcoxon signed-ranks test.

The average pH was significantly higher after the administration of famotidine than that after lansoprazole during the 1–2, 2–3 and 3–4 h study periods (median: 5.10 vs 3.10; *p* = 0.0069, 5.65 vs 3.25; *p* = 0.0217, 5.75 vs 2.85; *p* = 0.0208). No significant differences were observed in the 0–1, 4–5, and 5–6 h study periods. The average pH was significantly higher after the administration of vonoprazan than after lansoprazole during the 3–4, 4–5 and 5–6 h study periods (median: 5.20 vs 2.85; *p* = 0.0411, 6.00 vs 2.95; *p* = 0.0165, 5.00 vs 2.55; *p* = 0.0365). No significant differences were observed in the 0–1, 1–2 and 2–3 h study periods (Fig. 2).

### Holding time (%) of the various pH levels over the 6-h monitoring period

**Fig. 3:**
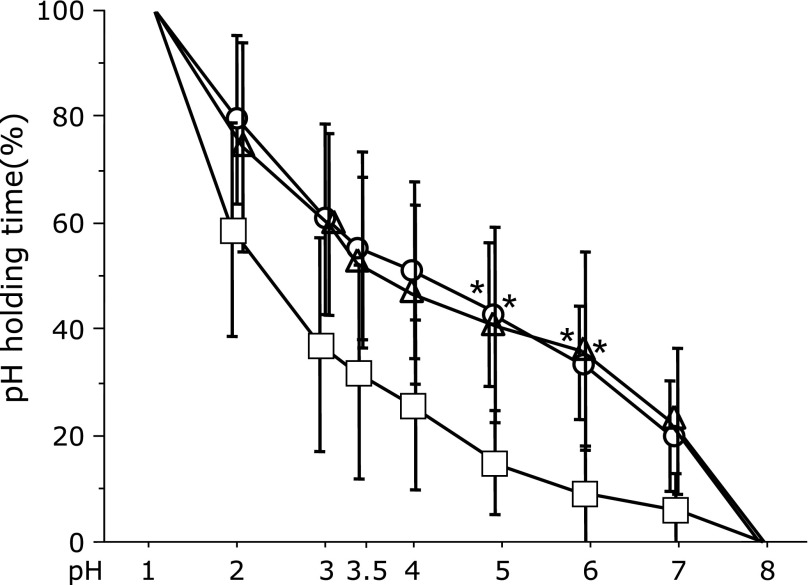
During the 6-h study period, famotidine (20 mg) and vonoprazan (20 mg) yielded a longer duration of pH >5 and 6 than did lansoprazole (30 mg). Circles (famotidine), triangles (vonoprazan) and squares (lansoprazole), mean values; vertical lines, SD; horizontal line, ±SD. *p<0.05 according to the Wilcoxon signed-rank test.

In the 6-h study period, the administration of famotidine caused a longer duration of pH >5 or 6 than did the administration of lansoprazole (median: 39.65% vs 10.6%; *p* = 0.0125, 35.3% vs 3.25%; *p* = 0.0125). Similar results were found for vonoprazan and lansoprazole (median: 45.6% vs 10.6%; *p* = 0.0367, 38.55% vs 3.25%; *p* = 0.0218) (Fig. 3).

## Discussion

In this study, we examined the change in the intragastric pH in the early stage after a single oral administration of vonoprazan, lansoprazole or famotidine in *HP*-negative subjects. Vonoprazan and famotidine had a significantly faster onset of action and caused a stronger suppression of gastric acid secretion than did lansoprazole.

Lansoprazole is a benzimidazole class of anti-secretory agents and the second approved PPI in Japan. Lansoprazole inhibits gastric acid H^+^/K^+^-ATPase in the gastric parietal cells forming part of the proton pump that performs acid secretion. Lansoprazole is widely used for treating reflux oesophagitis, peptic ulcer diseases such as gastric and duodenal ulcers, and maintenance treatment of erosive oesophagitis. Most PPIs, including lansoprazole, are metabolized and inactivated by CYP2C19 and CYP3A4 of the liver enzyme cytochrome P450. There are genetic polymorphisms of extensive CYP2C19 metabolizers and poor metabolizers in CYP2C19, and significant differences in plasma drug concentration have been observed. These factors affect the degree of inhibition of acid secretion.^(13–17)^

By contrast, famotidine is metabolized by the kidney and not the liver.^(18)^ Thus, the inhibition of acid secretion by famotidine is not affected by the CYP2C19 phenotype or genotype status.

Vonoprazan is a P-CAB that represents a new class of gastric-acid-suppressive agents. Vonoprazan was approved for use in *HP* eradication in addition to peptic ulcer and reflux esophagitis in Japan. Nishizawa *et al.*^(19)^ reported that with clarithromycin-based triple therapy, Vonoprazan is a better choice of antisecretory agent compared to PPIs, especially in young to middle-aged patients.

Vonoprazan is metabolized and inactivated by CYP3A4 of the liver enzyme cytochrome P450. A phase I study of vonoprazan in healthy male volunteers has revealed that it rapidly and strongly inhibits gastric acid secretion within 24 h after a single dose in the range of 20–120 mg.^(20)^ Furthermore, the pharmacokinetics of vonoprazan is not affected by the CYP2C19 genotype.^(20,21)^

The poor-metabolizer phenotype of CYP2C19 recognizes the difference in occurrence frequency among races. Poor metabolizers account for only 2% to 6% in Caucasians and 9% to 23% in Japanese individuals.^(22–24)^ We also confirmed genetic polymorphisms in our study; three of the 10 subjects (33.3%) were poor metabolizers. Possibly, the plasma drug concentration of lansoprazole was high in these three subjects, but in this study, there was no significant difference between poor metabolizers and extensive metabolizers regarding intragastric acidity.

Treatment with PPIs is known to be the most effective means of healing peptic ulcers. In addition, PPIs provide more effective and prompt ulcer healing than H_2_RAs, with respect to the alleviation of symptoms.^(25)^ However, our study demonstrated that H_2_RA increased the intragastric pH more rapidly than a conventional PPI did. Prior studies have shown that the repeated oral or intravenous administration of PPIs was effective in suppressing acid secretion. In addition, the stable effects of PPIs are achieved after approximately 5 days.^(26,27)^ Another study showed that a single initial dose of a PPI failed to achieve an intragastric pH >4 on the first day of treatment.^(28)^ However, a PPI was more effective than an H_2_RA in suppressing acid secretion in healthy volunteers, patients with duodenal ulcers and patients with GERD after 5 to 7 days of treatment.^(29,30)^ In an autoradiography study, H_2_RAs bound to all parietal cells uniformly, whereas PPIs bound only to young activated parietal cells. In the early period of drug treatment, the anti-secretory action of PPIs had a slower onset than that of H_2_RAs.^(31)^ It appears that the suppression of gastric acid secretion by H_2_RAs is superior to that by PPIs. However, H_2_RAs have disadvantages of a relatively short duration of action, the development of tolerance, and an incomplete suppression of acid secretion in response to diet.

Some of GEED patients are reported to be refractory to PPI therapy. Kawai *et al.*^(32)^ reported combination therapy with Rikkunshito and a PPI improves quality of life in patients with patients with PPI-refractory GERD. Studies for P-CAB, which strongly inhibits acid secretion than PPI, refractory GERD are also necessary in the future.

Patients receiving treatment at a therapeutic dose of conventional PPIs sometimes experience insufficient gastric acid inhibition at night. This phenomenon is generally called nocturnal acid breakthrough (NAB) and is defined as a stomach pH of <4 for over 1 h. Acid reflux at night can worsen GERD symptoms that are improving. Therefore, suppression of persistent acid reflux during the night is considered important for alleviating symptoms.^(9)^ H_2_RAs are globally used to treat NAB, but it is known that the effects of H_2_RAs decrease with sequential administration.^(33)^

Most patients with mild GERD and infrequent symptoms use a PPI only when symptoms appear. On-demand therapy for patients with mild GERD after receiving initial therapy with PPIs improves the quality of daily life and is cost effective.^(34,35)^ The rapid acid suppression effect is important in on-demand therapy aiming at improving heartburn. Although many patients experience heartburn after meals,^(36–38)^ H_2_RAs are inappropriate for treatments to suppress acid secretion in response to diet because the condition of the stomach after meals slows the effectiveness of H_2_RAs.^(39)^

The main cause of GERD symptoms is believed to be acid reflux to the oesophagus. The long-term suppression of gastric acid control is necessary for GERD symptoms. However, the heartburn associated with mild GERD is a temporary symptom, mainly due to short-term gastric acid reflux. Therefore, rapid onset and long-term acid suppression play important roles in eliminating these symptoms.

Our results showed no significant differences between famotidine and vonoprazan. However, vonoprazan is characterized by not requiring activation by acid and is effective even when administered on an empty stomach. Therefore, vonoprazan is considered an agent useful for on-demand therapy and for patients with NAB because it can quickly suppress gastric acid secretion.

## Conclusions

Oral administration of vonoprazan and famotidine in *HP*-negative healthy male subjects caused the intragastric pH to rise more quickly than did lansoprazole. This result shows that vonoprazan may be suitable for initial therapy, on-demand therapy and patients with NAB.
