# Erratum

**DOI:** 10.3164/jcbn.17-128_Erratum1

**Published:** 2018-12-13

In the article by Ohkuma *et al*. (*Journal of Clinical Biochemistry and Nutrition* 2018; 63: 80–83) “Comparison of the early effects of vonoprazan, lansoprazole and famotidine on intragastric pH: a three-way crossover study,” an error appeared in the legends of Fig. 2 and 3. The error should be median and vertical line. And an error appeared in Fig. 2 and 3. The circles (famotidine), triangles (vonoprazan) and squares (lansoprazole) appeared in an incorrect place. This correction is limited to this column and does not the conclusions of the study. The corrected Fig. 2 and 3 with figure legends were shown as follows.

## Figures and Tables

**Fig. 2:**
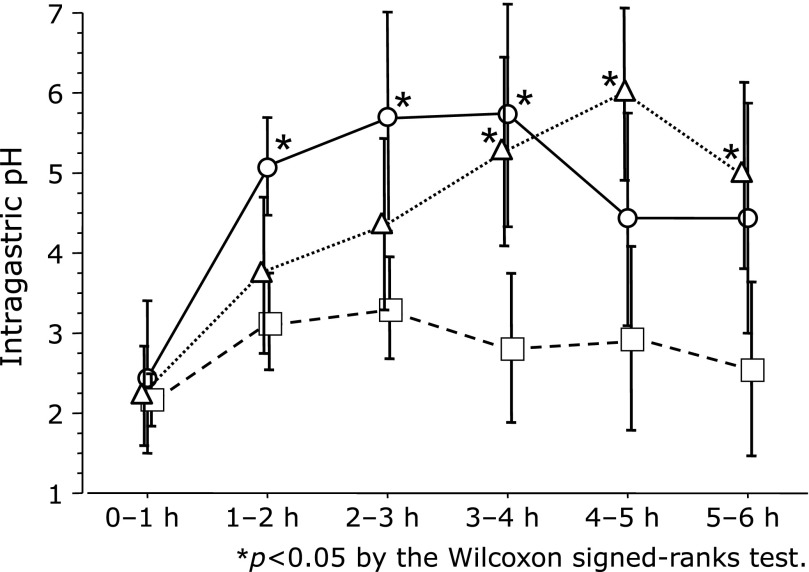
Famotidine (20 mg) resulted in a higher average pH than did lansoprazole (30 mg) in the 1–2, 2–3 and 3–4 h study periods after administration. Vonoprazan (20 mg) resulted in a higher average pH than did lansoprazole (30 mg) in the 3–4, 4–5 and 5–6 h study periods after administration. Circles (famotidine), triangles (vonoprazan) and squares (lansoprazole), median values; vertical lines, SD; vertical line, ±SD. *p<0.05 according to the Wilcoxon signed-ranks test.

**Fig. 3:**
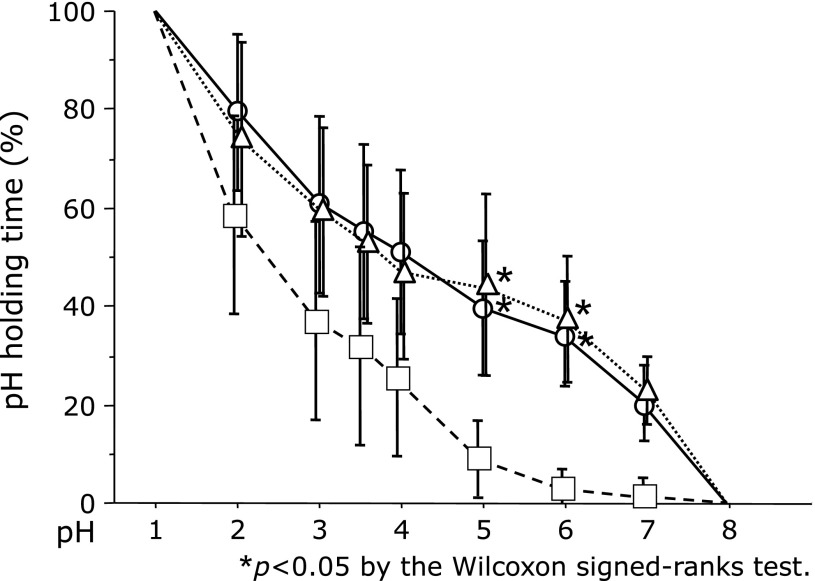
During the 6-h study period, famotidine (20 mg) and vonoprazan (20 mg) yielded a longer duration of pH >5 and 6 than did lansoprazole (30 mg). Circles (famotidine), triangles (vonoprazan) and squares (lansoprazole), median values; vertical lines, SD; vertical line, ±SD. *p<0.05 according to the Wilcoxon signed-rank test.

